# The corneal epitheliotrophic abilities of lyophilized powder form human platelet lysates

**DOI:** 10.1371/journal.pone.0194345

**Published:** 2018-03-16

**Authors:** Lily Wei Chen, Chien-Jung Huang, Wen-Hui Tu, Chia-Ju Lu, Yi-Chen Sun, Szu-Yuan Lin, Wei-Li Chen

**Affiliations:** 1 Department of Ophthalmology, National Taiwan University Hospital, Taipei, Taiwan; 2 Department of Ophthalmology, Taipei Tzu Chi General Hospital, The Buddhist Tzu Chi Medical Foundation, New Taipei City, Taiwan; 3 Department of Ophthalmology, Cathay General Hospital, Taipei, Taiwan; 4 Center of Corneal Tissue Engineering and Stem Cell Biology, National Taiwan University Hospital, Taipei, Taiwan; Georgetown University, UNITED STATES

## Abstract

**Purpose:**

To evaluate whether lyophilized human platelet lysate (HPL) powder can preserve the growth factor concentrations and epitheliotrophic properties of liquid HPL, and potentially be used as a clinically-friendly treatment option.

**Methods:**

Two commercialized liquid HPLs, UltraGRO TM (Helios, Atlanta, GA) and PLTMax (Mill Creek, Rochester, MI), were obtained and converted to lyophilized powder. After redissolution, lyophilized powder HPLs were compared with liquid HPLs, as well as human peripheral serum (HPS) and fetal bovine serum (FBS) in liquid or redissolved lyophilized powder forms. Concentrations of epidermal growth factor (EGF), transforming growth factor-β1 (TGF-β1), platelet-derived growth factor-AB (PDGF-AB) and platelet-derived growth factor-BB (PDGF-BB) were evaluated by enzyme-linked immunosorbent assay (ELISA). Human corneal epithelial cell line was incubated with the blood derivatives and evaluated for cell migration with scratch-induced directional wounding and proliferation with MTS assays. Cell differentiation was examined by transepithelial electrical resistance (TEER). Fluorescein staining and in vivo confocal microscopy were used to evaluate in vivo corneal epithelial wound healing in Sprague-Dawley rats that underwent corneal debridement and topical application of liquid and redissolved powder HPLs.

**Results:**

Liquid form and redissolved lyophilized powder form HPLs had similar concentrations of EGF, TGF-β1, PDGF-AB and PDGF-BB. *In vitro* experiments on cell migration, proliferation and differentiation and rat models on wound healing demonstrated no significant difference between the liquid and redissolved lyophilized powder forms for HPLs, HPS and FBS. *In vivo* confocal microscopy revealed similar wound healing process at different layers of cornea after corneal epithelial debridement between liquid form and redissolved lyophilized power form of HPLs.

**Conclusions:**

The redissolved lyophilized powder form of both commercialized HPLs showed similar growth factor concentrations and corneal epitheliotrophic abilities compared to the liquid form. Results suggest that the properties of liquid HPLs can be retained despite lyophilization and that lyophilized HPLs can be a treatment option for corneal epithelial disorders.

## Introduction

Human peripheral serum (HPS) shares several biochemical and biomechanical properties with natural tears and is known for its epitheliotrophic property, making it a favorable treatment option for ocular surface disorders such as persistent epithelial defects, dry eye syndrome, superior limbic keratoconjunctivitis and recurrent corneal erosions [1–13]. However, the preparation of HPS from the retrieval of patients’ peripheral blood to the manufacturing of eye drops, is often tedious and inconvenient for clinical use [14]. The stringent requirement for HPS to be stored under 0°C and the limited shelf life increase the difficulty for patients to use the product correctly [15]. Moreover, the lack of a standardized dilution protocol for the preparation of HPS brings into question the reproducibility of this treatment. Preparing HPS from peripheral blood samples of unhealthy patients may undermine the quality of HPS and the presence of proinflammatory agents in HPS may lead to unwanted side effects [16, 17].

Abundant growth factors and cytokines that are stored in platelet granules can be naturally released by thrombin activation [18–19] and clotting, or artificially released by freeze/thaw-mediated platelet lysis, sonication or chemical treatment [20]. Human platelet lysates (HPLs) prepared by the various release protocols are found to be suitable alternatives to fetal bovine serum (FBS) as culture supplements in cell therapy and tissue engineering, enabling efficient cultivation of human cells without the use of animal serum [21–26]. Mitogenic growth factors stored in HPLs include fibroblast growth factor (FGF), platelet-derived growth factor (PDGF), transforming growth factor (TGF) and epidermal growth factor (EGF) [27–29]. Recently, several studies demonstrate that HPL has the potential to promote corneal epithelial wound healing [30–33]. However, liquid HPLs have the drawbacks of a short shelf life and an inconvenient reliance on freezers for storage.

Our previous study showed that commercialized HPLs, UltraGRO TM (Helios, Atlanta, GA) and PLTMax (Mill Creek, Rochester, MI), have corneal epitheliotrophic abilities and wound healing rates similar to those of HPS and FBS both *in vivo* and *in vitro* [30]. These results suggested that commercialized HPL, with its more consistent product quality, could potentially replace HPS as a treatment for ocular surface disorders. In the present study, we aimed to understand how lyophilization of liquid HPLs into powder forms might affect corneal epitheliotrophic abilities. Like how milk powder can be conveniently stored and preserved for long periods of time, HPL powders may potentially overcome the storage limitations of liquid HPL and still be easily redissolved back to liquid forms for use in patients. The effects of powder HPLs on cellular proliferation, migration and differentiation after redissolution were compared with those of liquid HPLs in corneal epithelial cell line. A rat model was used to confirm the *in vivo* effects. We also compared the levels of several important corneal epitheliotrophic factors in liquid and powder forms of HPLs.

## Materials and methods

### Reagents and antibiotics

Dispase II was obtained from Roche Diagnostics Corporation (Indianapolis, IN). Phosphate-buffered saline (PBS), trypsin-EDTA, F12, Dulbecco’s modified Eagle’s medium (DMEM), amphotericin B and FBS were purchased from Gibco (Rockville, MD). Enzyme-linked immunosorbent assay (ELISA) kit for human EGF kit was purchased from eBioscience (San Diego, CA). ELISA kits for TGF-β1, PDGF-AB and PDGF-BB were acquired from RayBiotech, Inc. (Norcross, GA), R&D Systems (Minneapolis, MN) and PeproTech (Rocky Hill, NJ), respectively. All other reagents were obtained from Sigma-Aldrich (St. Louis, MO).

### Preparation of blood derivatives

#### Preparation of FBS

FBS was obtained from Gibco (Rockville, MD) and stored at -20°C in sterile tubes. Corneal epithelial cell line was cultured in DMEM with 3%, 5% and 10% FBS. For animal experiments, 20% FBS was prepared in Refresh Tear (Allergan, Inc. Parsippany, NJ).

#### Preparation of HPS

For the preparation of HPS, whole blood samples (50 ml each) were obtained from 10 healthy volunteers (mean age: 30.3 ± 10.2 years) via venipuncture. The blood samples were kept at room temperature (20–25°C) for 4 hours to clot before centrifugation at 3000g for 15 minutes. In order to remove unwanted immune complements, the blood serum was heated at 56°C for 30 minutes and carefully filtered into 10 ml aliquots in a sterile environment to be stored at -20°C. HPS was similarly diluted to 3%, 5% and 10% concentrations with DMEM and 20% concentration with Refresh Tear (Allergan, Inc) for the *in vitro* and *in vivo* experiments. The protocol was approved by the Institutional Review Board for Human Studies at the National Taiwan University Hospital (201510123RINB). All volunteers reported no history of chronic diseases and were not taking any medications.

#### Preparation of HPLs

Two commercialized liquid HPLs, UltraGRO TM (Helios, Atlanta, GA) and PLTMax (Mill Creek, Rochester, MI), were stored at -20°C in sterile tubes. HPLs were diluted to 3%, 5% and 10% concentrations in DMEM for *in vitro* experiments and 20% concentration in Refresh Tear for *in vivo* experiments.

#### Lyophilization of the blood products and redissolution into liquid forms

Liquid blood derivatives (HPLs, HPS, FBS) in volumes of 50ml each were placed in buffer with trehalose, and put through a programmed freeze-dry process via the LyoStar II lyophilizer (FTS Systems, Stone Ridge, NY, USA) with a freezing point of—60°C in vacuum overnight. The resulting lyophilized powder for each blood product was stored at room temperature for up to 3 months, and then dissolved in double distilled water to reconstitute into a solution with the original volume (50 ml) prior to use in the *in vitro* and *in vivo* experiments.

### Quantification of epitheliotrophic factors

Epitheliotrophic factors were quantified in the liquid and lyophilized forms of 3 different human blood derivatives (HPS and 2 HPLs) using a modification of the method previously used by Shen, et al. [13]. The concentrations of EGF, TGF-β1, PDGF-AB, and PDGF-BB were measured using ELISA.

### Culture of human corneal epithelial cell line (HCEC)

Cells of the human corneal epithelial cell line (CRL-11515) from ATCC were centrifuged and resuspended in DMEM-F12 medium supplemented with antibiotic-antimycotic agents (100μg/ml penicillin/streptomycin and 1.25μg/ml amphotericin B). Different concentrations (3%, 5%, 10%) of liquid and redissolved lyophilized powder forms of each blood derivative (HPS, HPLs and FBS) were added to the culture medium, which was replaced every 2–3 days. We used only cells from passages 2–3 of the initial culture. Cells cultured without any serum were used as control group.

### Cell-migration: Scratch-induced directional wounding assay

HCECs (4 x 10^5^ cells/ml) were cultivated in 12-well tissue culture plates and maintained in media with 5% blood derivatives (HPLs, HPS and FBS) that were made from liquid and redissolved lyophilized powder forms. When cells reached confluency, a 200μl micropipette tip was used to create a 1mm linear scrape (“wound”) across the tissue. Degrees of “wound” closure were recorded at 0, 8, 12 and 16 hours after injury using a digital camera mounted on an inverted microscope (Diagnostic Instruments, Inc., Sterling Heights, MI). The average residual gap between migrating cells of opposing “wound” edges was measured with an image analysis program (Image J 1.37v; Wayne Rasband at the Research Services Branch, National Institute of Mental Health, Bethesda, MD). “Wound” closure was quantified using the wound healing ratio, which was calculated by taking the difference between the initial and current cell-free areas, and dividing that by the initial cell-free area. All experiments were replicated six times to ensure consistent results.

### Cell proliferation: MTS assay

HCECs (5 x 10^3^ cells/well) were seeded into a 96- well plate and cultured for 3 days with different concentrations (3%, 5%, 10%) of blood derivatives (HPLs, HPS, FBS) that were made from liquid and redissolved lyophilized powder forms. The number of viable cells were quantified using the MTS assay (Promega Corp., Madison, WI) after incubating for 24, 48 and 72 hours. As only viable cells had the mitochondria to reduce 3-(4,5-dimethylthiazol-2-yl)-5-(3-carboxy-methoxyphenyl) -2-(4-sulfophenyl)-2H-tetrazolium inner salt (MTS) into soluble formazan, the number of viable cells was proportional to the amount of formazan produced. Cellular proliferation was quantified after determining the formazan concentrations with an ELISA microplate reader (Model ELx 800; Bio-TEK Instruments, Inc., Winooski, VT) by measuring the absorbances at 290 nm (test wavelength) and 650 nm (reference wavelength). Control wells contained culture medium without cells. All experiments were replicated six times to ensure consistent results.

### Cell differentiation: Measurement of transepithelial electrical resistance (TEER)

TEER was used to determine cell differentiation and function. HCECs (1 x 10^5^ cells/well) were seeded in the upper chamber of a Costar transwell (Corning Costar, Cambridge, MA; 1.12 cm^2^ diameter, 0.4μm pore size) and cultured for 3 days in 5% blood derivatives (HPLs, HPS and FBS) that were made from liquid and redissolved lyophilized powder forms. Millicell-ERS electrical resistance system (Millipore, Bedford, MA) was used to measure the electrical resistance (Ω) of the transwell filter membrane after the cells reached full confluency. The resistance value was multiplied with the surface area (1.12cm^2^) of the monolayer of cells to yield TEER (Ωcm^2^). All experiments were replicated six times to ensure consistent results.

### Corneal epithelial wound healing: A rat model

Male Sprague-Dawley rats, aged 16–24 weeks, were anesthetized with intramuscular injections of tiletamine/zolazepam (6.25mg/kg) (Zoletil; VIRBAC, Carros, France) and xylazine (5.83mg/kg) (Rompun; Bayer Korea Ltd. Gyeonnggi, Korea). Topical proparacaine (Alcaine; Alcon Laboratories, Inc., Fort Worth, TX) was applied to each eye. A trephine (4mm in diameter) was used to mark the central cornea before debriding the corneal epithelium with a corneal rust ring remover with a 0.5mm-burr (Algerbrush IITM; Alger Equipment Co., Inc., Lago Vista, TX) under the operating microscope (OPMI Pico I; Carl Zeiss Meditec, Jena, Germany). Liquid and redissolved powder forms of HPLs in 20% concentrations were applied over a 48-hour period (2 cycles of 12 hours with and 12 hours without topical eye drops). Corneal epithelial defects were stained with fluorescein and photographed under an operating microscope at 0, 12, 24 and 48 hours. Wound healing ratio was determined as the difference between the initial and current epithelial defect areas, divided by the initial epithelial defect area. All experiments were replicated six times to ensure consistent results.

All animals used in this study were handled according to the ARVO Statement for the Use of Animal in Ophthalmic and Vision Research and the protocol was approved by the Animal Care and Use Committee of National Taiwan University. No animal died, appeared ill or suffered greatly prior to the experimental endpoints, although a protocol was in place for early humane endpoints in cases where animals appeared irritable or in severe pain. Animals were maintained on a 12:12-hr light/dark cycle, and food and water were available *ad libitum*.

### *In vivo* confocal microscopy of corneal epithelial morphology

Male Sprague-Dawley rats, aged 16–24 weeks, underwent anesthesia and corneal debridement as described above. HRT3 confocal microscope (Heidelberg Engineering GmbH, Heidelberg, Germany) was used to view corneal epithelium and superficial stroma at 100 um underneath the corneal basal epithelium with image dimensions of 400×400 μm^2^ and transverse resolution of 1 μm. Rats (n = 2 for each treatment) were given liquid and redissolved powder forms of UltraGRO diluted to 20% in Refresh Tear each hour for 48-hour period (2 cycles of 12 hours with and 12 hours without topical eye drops) and photographed with confocal microscopy 1 week after debridement. Rats without debridement were used to photograph normal corneal epithelium for comparison. Rats that underwent debridement but no topical treatment were used as control.

### Data evaluation and statistical methods

All data were analyzed with ANOVA and Student’s t-test for statistical significance (p< 0.05).

## Results

### Appearance of blood derivatives

**Fig 1:**
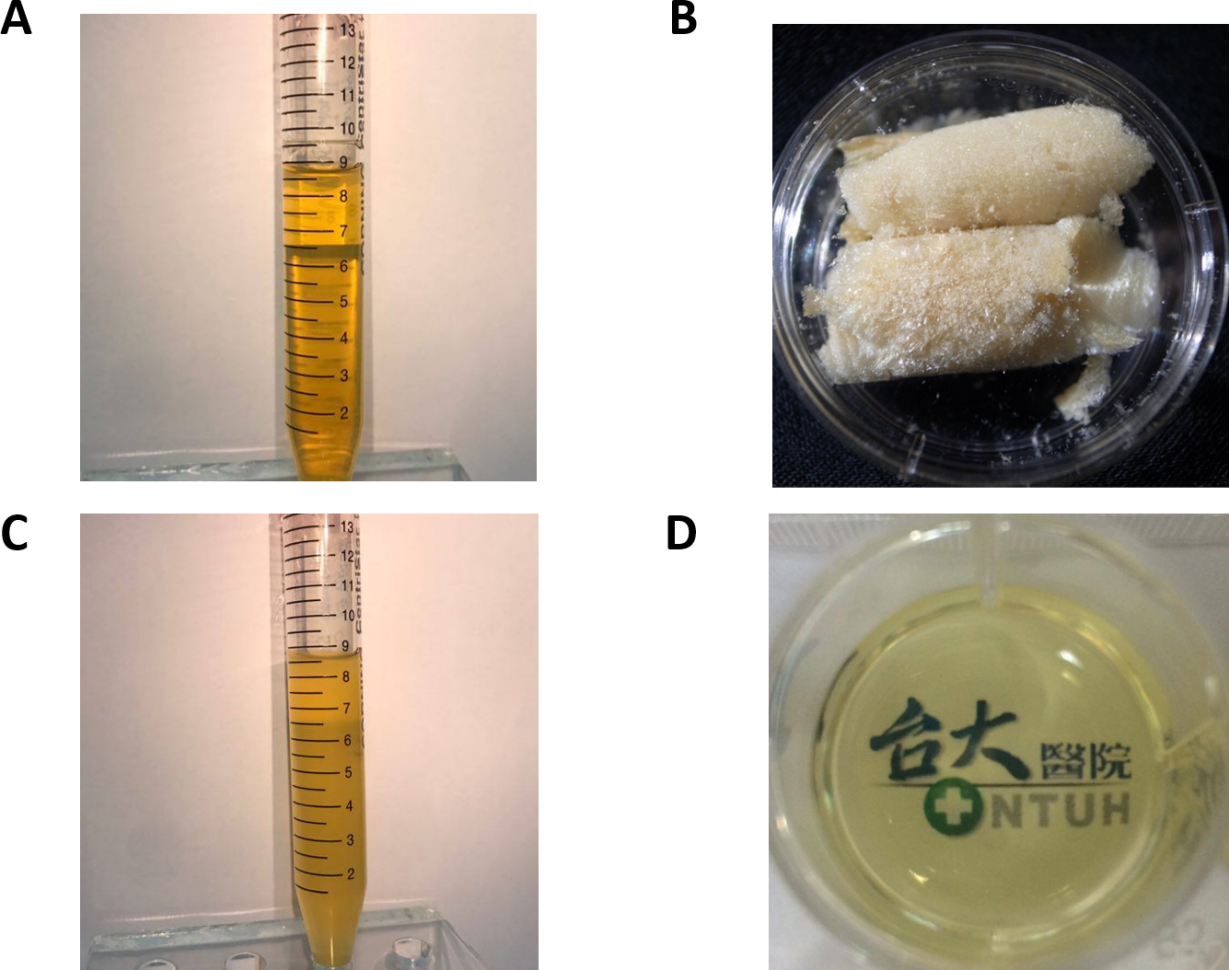
Appearance of various forms of HPS. (A) liquid form. (B) lyophilized powder form. (C) redissolved lyophilized powder form in plastic tube. (D) redissolved lyophilized powder form in plastic well of a 12-well plate (volume of 1ml). Redissolved lyophilized powder form HPS showed clear, faint yellow color with satisfactory transparency.

**Fig 2:**
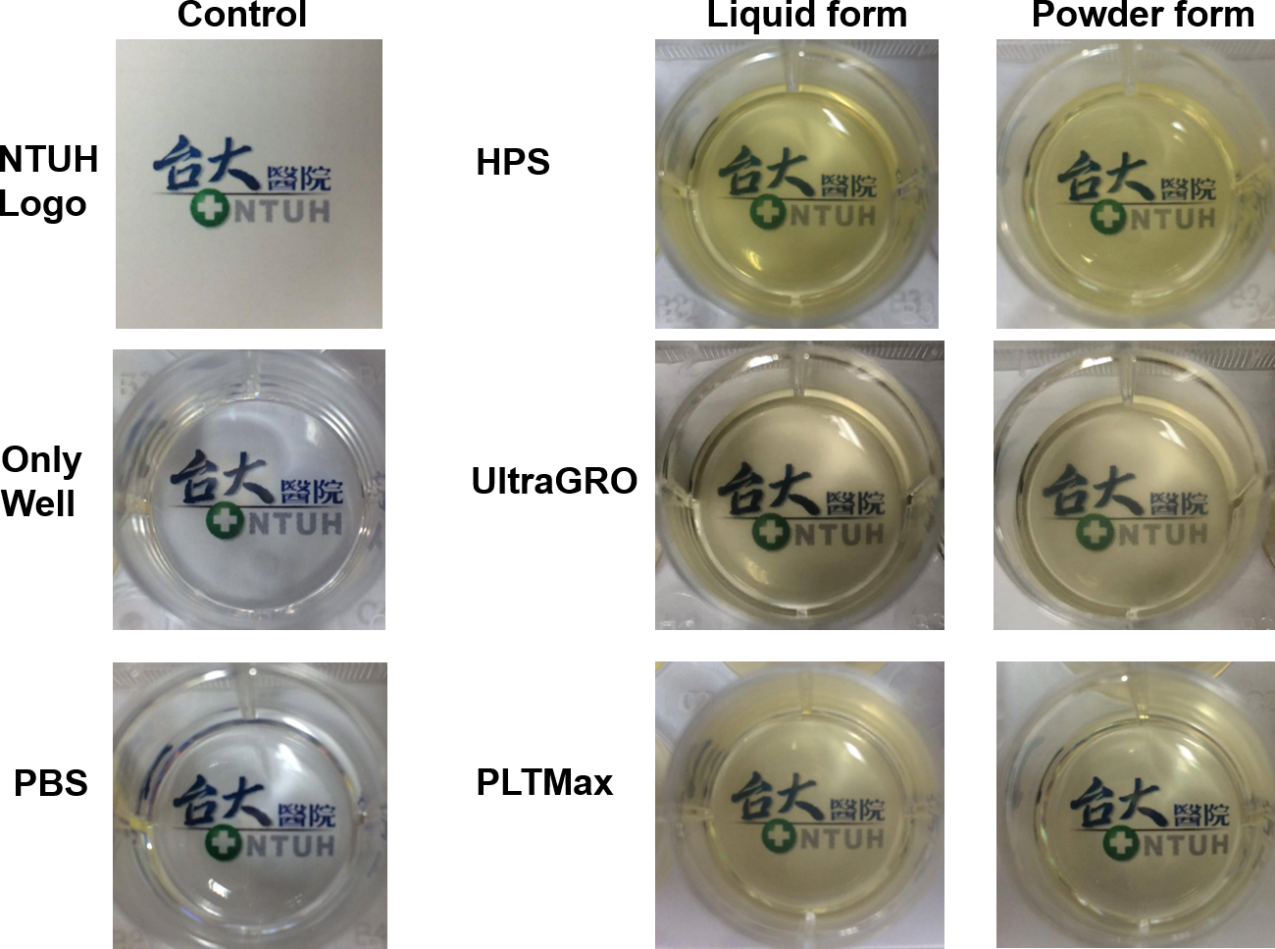
Comparison of liquid and redissolved lyophilized powder forms of HPS and HPLs. Control column on the left shows the NTUH (National Taiwan University Hospital) logo, plastic well, and plastic well containing PBS. HPS and HPLs (UltraGRO and PLTMax) shown on the right appear as clear yellow solutions with similar levels of transparency between the the liquid and redissolved lyophilized powder forms.

Fig 1 shows the various forms of HPS. The clear yellow appearance of liquid HPS (Fig 1A) was retained after lyophilization into powder form (Fig 1B) and redissolution with distilled water to the original volume (Fig 1C and 1D). The appearance of the two commercialized HPLs, UltraGRO and PLTMax, were also similar between the original liquid forms and the redissolved lyophilized powder forms (Fig 2).

### Quantification of epitheliotrophic factors

**Fig 3:**
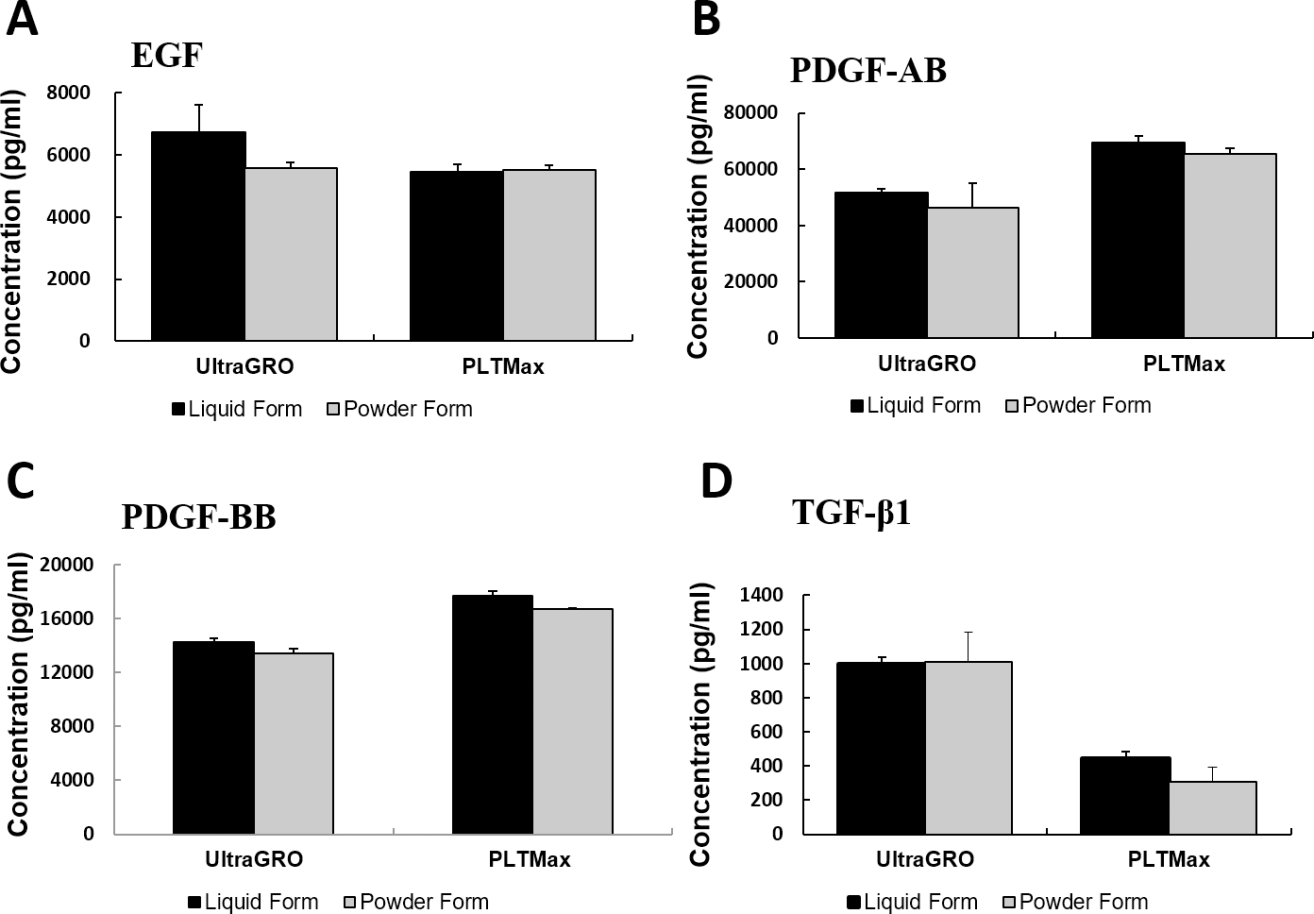
Quantification of epitheliotrophic factors in liquid and redissolved lyophilized powder forms of HPLs with the ELISA assay. Concentrations of (A) EGF, (B) PDGF-AB, (C) PDGF-BB and (D) TGF-β1 were measured in UltraGRO and PLTMax. There were no statistically significant differences in the concentration levels between liquid and redissolved powder forms (p>0.05). Error bars indicate SD.

Quantification of epitheliotrophic factors in liquid HPLs and the redissolved lyophilized powder HPLs was performed using the ELISA assay in UltraGRO and PLTMax (Fig 3). There were no statistically significant differences in the concentrations of EGF, PDGF-AB, PDGF-BB and TGF-β1 between the liquid form and the redissolved lyophilized powder form of HPLs (p>0.05).

### Cell migration: Scratch-induced directional wounding assay

**Fig 4:**
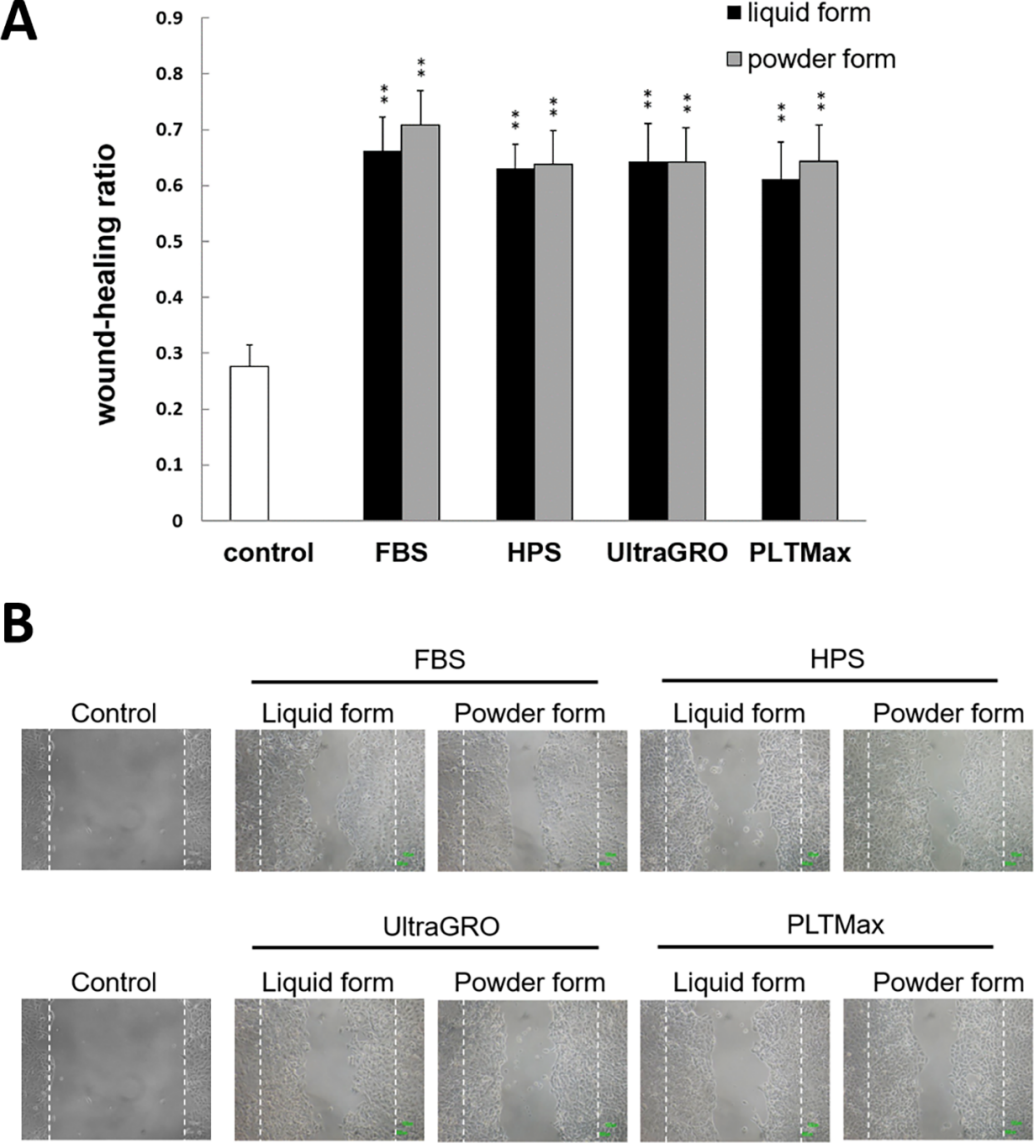
Scratch-induced directional wounding assay at 16 hours. (A) HCECs were cultured in 5% blood derivatives (FBS, HPS, UltraGRO and PLTMax) in liquid or redissolved lyophilized powder forms, and tested for wound-healing after scratching. All blood derivatives had increased wound-healing ratios compared to the control (serum-free) at 16 hours (p<0.01). There were no statistically significant differences in wound healing ratios between liquid and redissolved powder forms (p>0.05). **p<0.01 compared to the control. Error bars indicate SD. (B) Representative images from inverted microscopy that was done to evaluate wound healing in HCECs cells cultured in 5% blood derivatives (FBS, HPS, UltraGRO and PLTMax) in liquid and redissolved lyophilized powder forms. Control represents the original scraping area at 0 hours after injury.

Fig 4A demonstrates the wound healing ratios of HCEC at 16 hours after scraping. Cells were incubated with 5% of FBS, HPS, or HPLs (UltraGRO and PLTMax) in original liquid or redissolved lyophilized powder forms. Cells without blood derivatives were used as control. The healing ratios for liquid forms were 0.66±0.06, 0.63±0.04, 0.64±0.07, and 0.61±0.07 in FBS, HPS, UltraGRO, and PLTMax, respectively. The healing ratios for redissolved lyophilized powder forms were 0.71±0.06, 0.64±0.06, 0.64±0.06, and 0.64±0.07 in FBS, HPS, UltraGRO and PLTMax, respectively. All blood derivatives showed statistically significant increases in wound-healing ratios compared to the control (p<0.01). No statistically significant differences were noted between the liquid and the redissolved powder forms (p>0.05). Representative images taken with inverted microscopy are shown in Fig 4B.

### Cell proliferation: MTS assay

**Fig 5:**
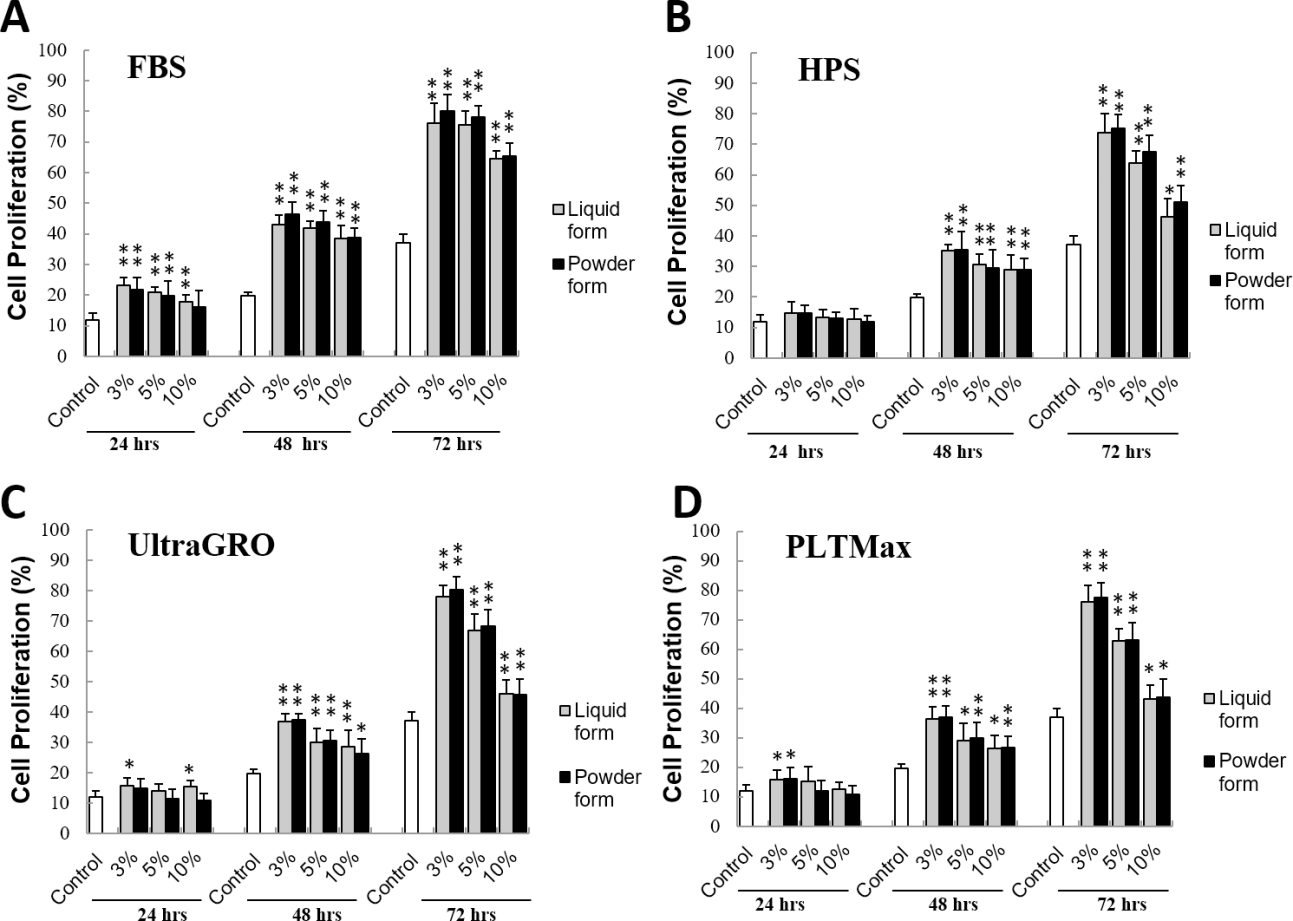
MTS assay to evaluate cell proliferation. HCEC were cultured in blood derivatives (FBS, HPS, UltraGRO and PLTMax) at 3%, 5% and 10% concentrations and tested with the MTS assay at 24, 48, and 72 hours. Compared to the control (no serum), cell incubated in blood derivatives resulted in increased proliferation at 48 and 72 hours. Liquid and redissolved lyophilized powder forms produced similar results at almost all concentrations and time points (p>0.05). *p<0.05 compared to the control.**p<0.01 compared to the control. Error bars indicate SD.

Fig 5 demonstrates the results of the MTS assay. HCEC numbers were fairly similar at 24 hours, but cells that were cultured in FBS, HPS, UltraGRO or PLTMax showed significantly increased proliferation rates at 48 and 72 hours compared to the control cells that were cultured in serum-free medium. There were no statistically significant differences between the liquid and redissolved powder forms of the blood derivatives for tested concentrations and time points, except for 10% UltraGRO at 24 hours. Blood derivatives at 3% concentration resulted in greater cell proliferation rates compared to 5% and 10% concentrations. Ten percent blood derivatives gave the lowest proliferation rates compared to other tested percentages of blood derivatives.

### Cell differentiation: Transepithelial electric resistance (TEER)

**Fig 6:**
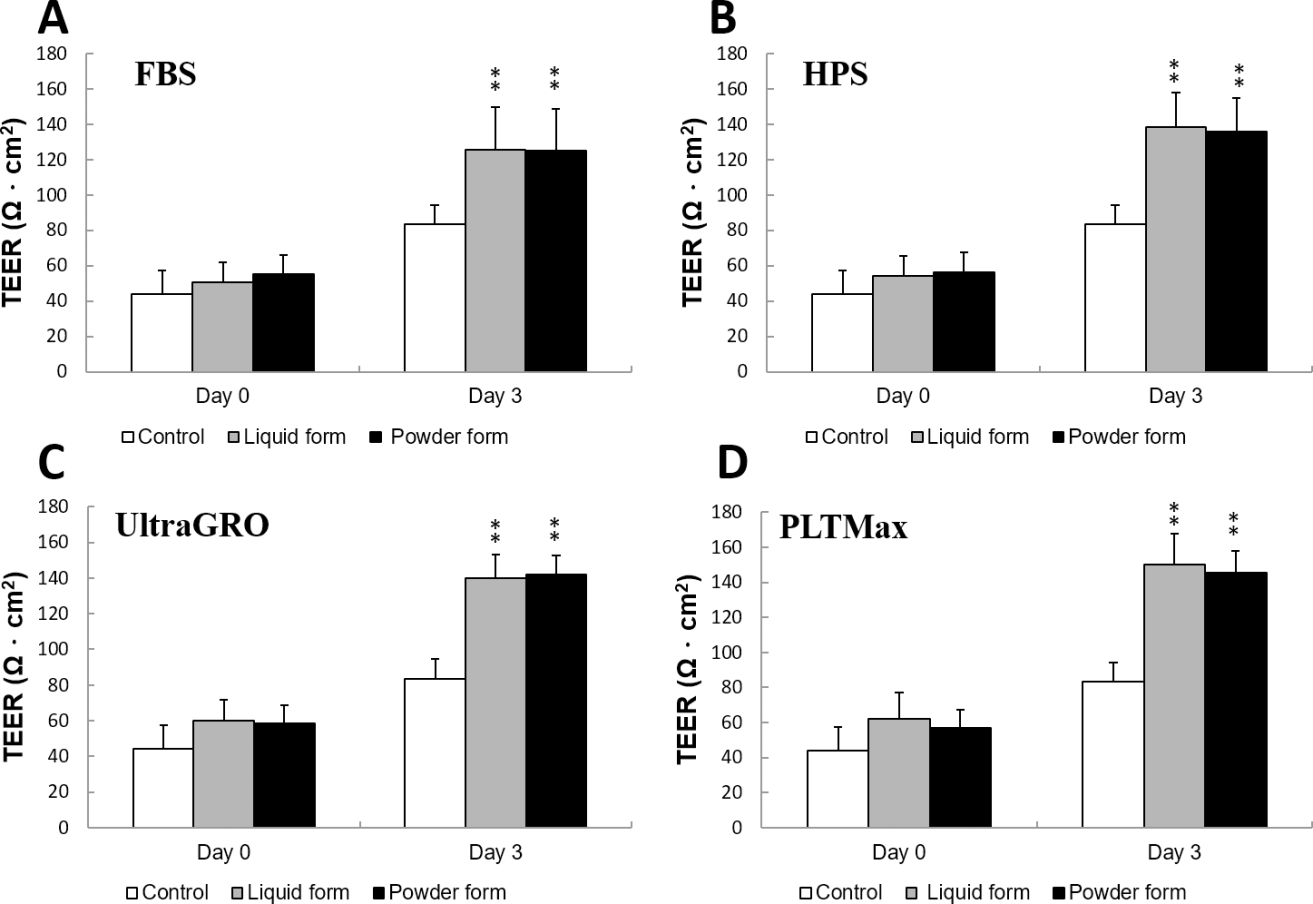
TEER assay to evaluate cell differentiation and function. HCEC were cultured in 5% blood derivatives (liquid and redissolved lyophilized powder forms of FBS, HPS, UltraGRO and PLTMax) and measured for TEER values on day 3. Compared to the control (no serum), cell incubated in blood derivatives produced increased TEER values. Liquid and redissolved lyophilized powder forms gave similar results (p>0.05). **p<0.01 compared to the control. Error bars indicate SD.

After incubating HCECs with 5% blood derivatives for 3 days, cellular differentiation was evaluated with TEER (Fig 6). Results of TEER were similar between the liquid and redissolved lyophilized powder forms (p>0.05). Cells cultured with blood derivatives had greater TEER values compared to the control cells, which reflected greater epithelial tightness and functional cell integrity (p<0.01).

### Corneal epithelial wound healing: A rat model

**Fig 7:**
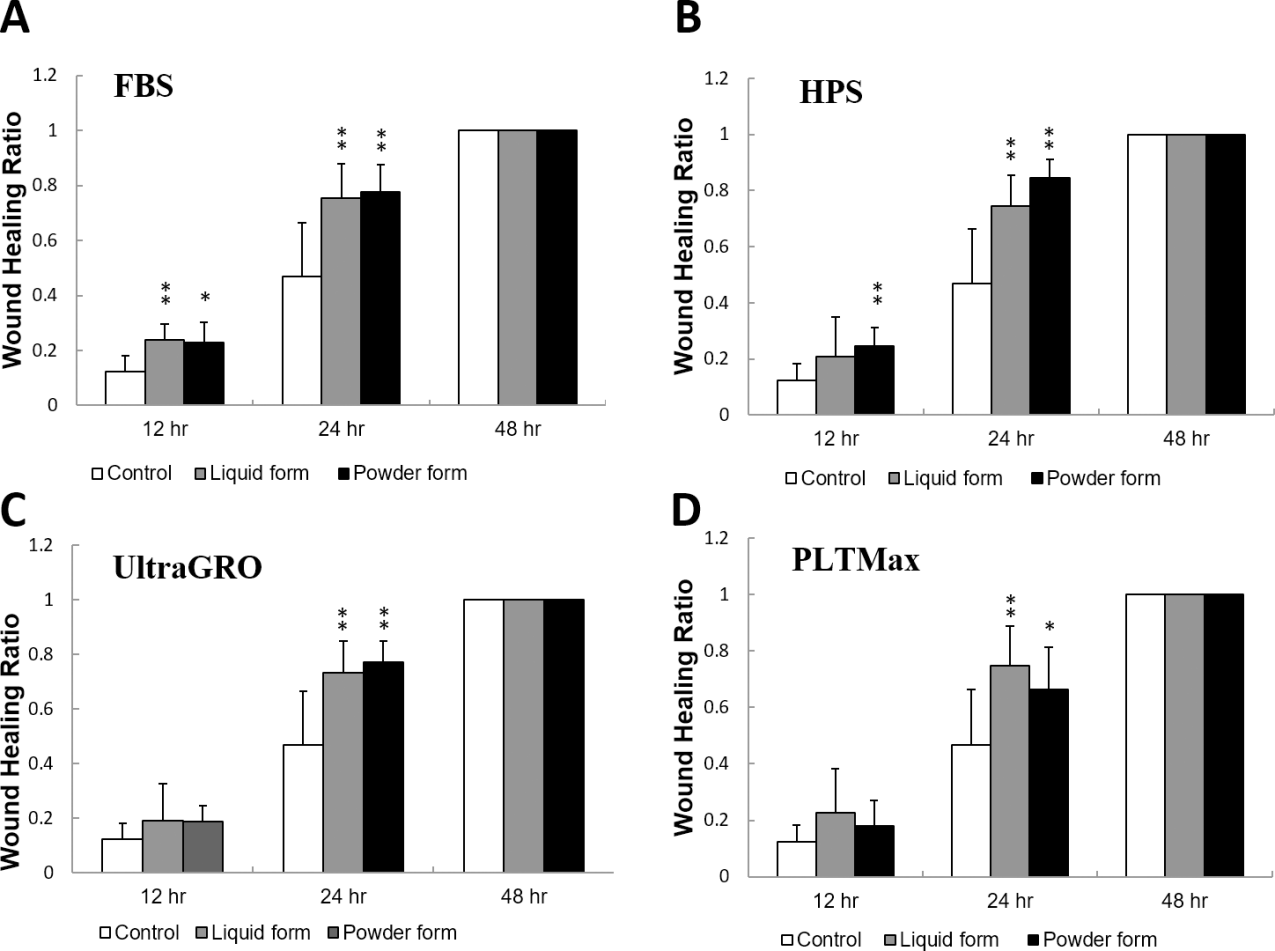
Corneal epithelial wound healing in rats. Rats underwent corneal epithelial debridement and topical applicaton of liquid or redissolved powder forms of blood derivatives. Corneal epithelial defects were stained with fluorescein and photographed at 0, 12, 24 and 48 hours to determine wound healing ratios. Compared to the control (no topical treatment), rats treated with blood derivatives had greater wound healing ratios at 24 hours. Effects were similar for liquid and redissolved powder forms. All rats had fully healed corneal epithelium at 48 hours. *p<0.05 compared to the control. **p<0.01 compared to the control. Error bars indicate SD.

Fig 7 demonstrates the *in vivo* rat corneal epithelial wound healing after epithelial debridement and topical treatment with Refresh Tear that contained 20% of liquid or redissolved powder form blood derivatives. Wound healing ratios were determined with fluorescein staining and compared to the control that had no treatment with blood derivatives. At 12 hours, rats given liquid form FBS, redissolved powder form FBS or redissolved powder form HPS had greater wound healing ratios compared to the control (p<0.01 or p<0.05). All blood derivatives, in liquid or redissolved powder forms, produced greater wound healing at 24 hours compared to the control (p<0.01 or p<0.05). There were no statistically significant differences between liquid and redissolved powder forms. At 48 hours, all rats had fully healed corneal epithelium with wound healing ratios of 1.

### *In vivo* confocal microscopic examination of corneal epithelial and stromal wound healing

**Fig 8:**
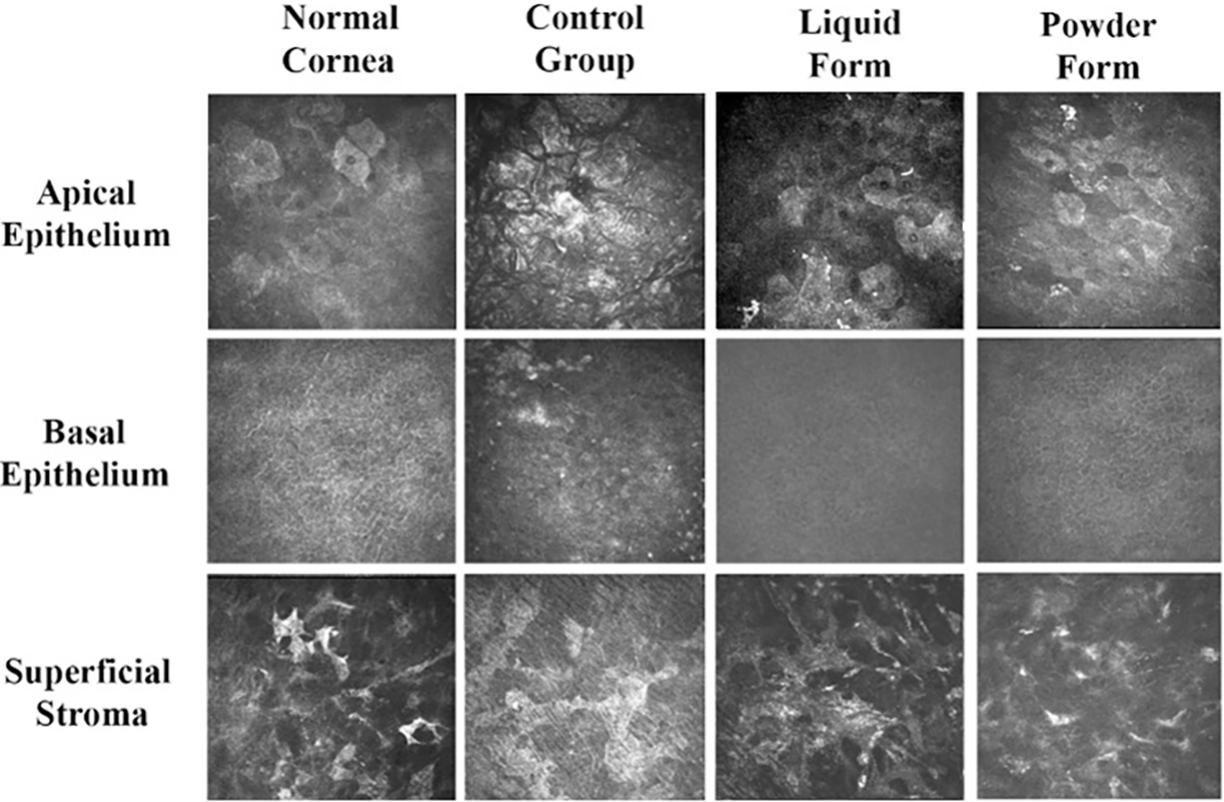
Cell morphologies during corneal epithelial wound healing in rats. Images were taken at 48 hours with a HR3 confocal microscope to view corneal epithelium for rats that underwent no corneal epithelial debridement (normal cornea), rats that underwent debridement but no treatment (control group), and rats that underwent debridement and treatment with UltraGRO (liquid form and powder form). Corneal epithelial debridement treated with UltraGRO had healed apical and basal epithelia similar to those of normal cornea, while the control had dry apical squamous cells and WBC infiltrates in the basal epithelium. The superficial stroma was not damaged, and appeared similar across the different groups. Image dimensions of 0.4mm x 0.4mm.

Similar to the *in vivo* wound healing assay with fluorescein staining, rats were given corneal debridement and treatment with Refresh Tear that contained 20% liquid form or redissolved powder form UltraGRO and then imaged with in vivo confocal microscopy. Fig 8 demonstrates the morphologies of the apical epithelium, basal epithelium and superficial stroma at 48 hours after wound healing. Rat corneas that were treated with either form of UltraGRO after debridement had healed apical and basal epithelia similar to those of rat corneas that had not been wounded. In contrast, the control group rat corneas that were debrided but given no topical treatment had dry squamous cells in the apical epithelium and infiltrates of inflammatory cells were found in the basal epithelium at the end of the experimental period.

## Discussion

Corneal epithelialization is an important clinical issue in many ocular surface disorders, including dry eye syndrome, recurrent corneal erosion, neurotrophic ulcer and limbal insufficiency [34–37]. Blood-derived products that contain large amounts of epitheliotrophic growth factors have been used as topical eye drops for treating corneal epithelial problems [14, 38–39]. HPS, cord blood serum and HPL are among such products. Of these, HPL demonstrated great clinical potential and has garnered much attention in recent years.

Under normal physiological activation of platelets, growth factors are released from intracellular alpha granules and thought to assist with wound healing [33]. There exist several well-known platelet growth factors, including epidermal growth factor (EGF), platelet-derived growth factor (PDGF), transforming growth factor -β1 (TGF-β1), fibroblast growth factor (FGF), and vascular endothelial growth factor (VEGF) [40–41]. Wound healing is a complex process and unlikely to be mediated by only one agent. A combination of platelet growth factors may provide a better way for clinical usage and epithelial healing. HPL, which is derived from blood and contains high levels of platelet growth factors, may thus be important for providing this need.

Commercialized blood derivatives have different forms and preparations. In addition to simple liquid products, there is fibrin glue (e.g. Tisseel Duo, Baxter) that is designed as a duo preparation with one syringe containing fibrinogen and the other syringe containing thrombin so that fibrin is formed with mixing when the product is ready to be used [42–43]. Lyophilized powder blood products, such as snake antivenoms, lyophilized fibrinogen, lyophilized plasma and lyophilized erythropoietin alpha have also been manufactured and proven to be user-friendly due to their stability at high temperatures and ease of use in austere conditions [44–46]. Liquid HPL is the only form of HPL that is commercially available, but has the drawbacks of a short shelf life and difficulty in transportation and storage due to temperature restrictions [15]. A different form of HPL may be more useful for commercializing HPLs and making these more convenient for patient use. We thus aimed to develop HPLs into a powder form that could be redissolved back to liquid HPLs right before clinical use.

Our previous study showed that the two commercialized HPLs (PLTMax and UltraGRO) contained significantly higher concentrations of EGF, TGF-β1, PDGF-AB, and PDGF-BB compared to HPS [30]. In this study, we tested the levels of these epitheliotrophic factors in the HPLs and found that lyophilization did not alter the concentration levels between the liquid and redissolved powder forms. These epitheliotrophic factors were chosen due to their critical roles in promoting corneal wound healing [47–48]. EGF can be secreted by lacrimal glands and corneal epithelial cells to induce corneal epithelial cell proliferation, TGF-β1 inhibits proliferation but stimulates cell differentiation and migration, while PDGF isoforms have been shown to stimulate corneal epithelial migration [32, 49–52].

In this study, we also compared the corneal epithelitrophic abilities of liquid and redissolved powder forms of HPLs, HPS and FBS. In the corneal epithelial cell line, there was no difference in cell migratory ability and differentiation ability by wound healing assay and TEER between the two forms of blood derivatives. There was no difference in cell proliferative ability between the liquid form and the redissolved powder form for most tested concentrations and time points. Additionally, these assays demonstrated increased cell proliferation, migration and differentiation for cells treated with blood derivatives. The *in vivo* models similarly demonstrated faster wound healing for rats treated with topical blood derivatives. These findings support the hypothesis that the growth factors in solutions of redissolved powder form HPLs not only retain their original concentrations but also their biochemical properties. We also found that redissolved HPL powders appeared as clear yellow solutions and retained the transparent property of commercialized liquid HPLs. When making powder HPLs into eye drops in the future, the levels of transparency in the redissolved solutions can quickly serve as an indication of HPL quality.

Human blood products like HPLs have fewer immunogenic risks in patients when compared with animal products [53]. The quality and safety tests for blood collection are well-established in most developed countries, where blood products are manufactured under the principles of Good Manufacturing Practice (GMP). In addition, the World Health Organization (WHO) guidelines encourage GMP implementation in blood establishments at a global level, thus increasing the availability of qualified sources [54]. Several recent studies on the potential of commercialized HPLs showed that HPLs could be used in the treatment of corneal epithelial disorders [30, 32–33]. Commercialized HPLs can be produced in bulk to meet patient demand and be supplied directly as eye drops in liquid or powder forms. This obviates the need to draw blood from patients and process human peripheral serum, which requires several hours of precipitation followed by centrifugation to obtain high concentrations of epitheliotrophic factors. Patients who need long-term use of human peripheral serum eye drops, such as those with Stevens-Johnson syndrome, bullous pemphigoid or severe graft-versus-host-disease, can especially benefit from a shorter waiting time and the lack of frequent blood draws. While the liquid HPLs have drawbacks of a short shelf life and stringent storage temperature requirements, lyophilized powder HPLs can overcome these limitations and be easily redissolved with water back into liquid eye drops before patient use. The lyophilized powder HPLs in our assays were stored for about 3 months prior to use, but longer storage times could potentially be tested in future studies.

This study adds to our previous study which examined the corneal epitheliotrophic effects of commercialized liquid HPLs and HPS. We found that growth factors and wound healing effects were retained despite lyophilization of HPLs into powder form and that lyophilization could potentially be used to produce HPL powder for clinical use. However, there were some limitations in our study. Firstly, the complexities of the tear film and corneal epithelium in humans cannot be fully presented with cell culture models. Secondly, the human corneal epithelial cell line was immortalized with SV-40 virus and could act differently from normal human corneal epithelium. Thirdly, a GMP laboratory was needed to produce the powder form of HPLs, and such production may be difficult to conduct in the hospital or regular laboratory. Pharmaceutical companies may need to transform liquid HPLs into powder forms in their GMP laboratories before distributing these to patients for redissolution into eye drops. *In vitro* experiments using primary cultivated corneal epithelial cells, additional *in vivo* studies, and possibly clinical trials may be needed to support the results.

In conclusion, powder form HPLs demonstrated similar corneal epitheliotrophic abilities as liquid HPLs after redissolution. The therapeutic effects of HPL powder may be comparable to those of autologous HPS and liquid HPL in the treatment of ocular surface disorders.

## References

[1] Noda-Tsuruya T, Asano-Kato N, Toda I, Tsubota K. Autologous serum eye drops for dry eye after LASIK. Journal of refractive surgery. 2006;22(1):61–6. 16447938 10.3928/1081-597X-20060101-13

[2] Hartwig D, Herminghaus P, Wedel T, Liu L, Schlenke P, Dibbelt L, et al Topical treatment of ocular surface defects: comparison of the epitheliotrophic capacity of fresh frozen plasma and serum on corneal epithelial cells in an *in vitro* cell culture model. Transfusion medicine. 2005;15(2):107–13. doi: 10.1111/j.0958-7578.2005.00559.x 15859976 10.1111/j.0958-7578.2005.00559.x

[3] Brown SM, Bradley JC. The effect of autologous serum eye drops in the treatment of severe dry eye disease: a prospective randomized case-control study. American journal of ophthalmology. 2005;140(3):565; author reply -6. doi: 10.1016/j.ajo.2005.03.067 16139023 10.1016/j.ajo.2005.03.067

[4] Matsumoto Y, Dogru M, Goto E, Ohashi Y, Kojima T, Ishida R, et al Autologous serum application in the treatment of neurotrophic keratopathy. Ophthalmology. 2004;111(6):1115–20. doi: 10.1016/j.ophtha.2003.10.019 15177961 10.1016/j.ophtha.2003.10.019

[5] Vajpayee RB, Mukerji N, Tandon R, Sharma N, Pandey RM, Biswas NR, et al Evaluation of umbilical cord serum therapy for persistent corneal epithelial defects. The British journal of ophthalmology. 2003;87(11):1312–6. 14609821 10.1136/bjo.87.11.1312 PMC1771905

[6] Ogawa Y, Okamoto S, Mori T, Yamada M, Mashima Y, Watanabe R, et al Autologous serum eye drops for the treatment of severe dry eye in patients with chronic graft-versus-host disease. Bone marrow transplantation. 2003;31(7):579–83. doi: 10.1038/sj.bmt.1703862 12692625 10.1038/sj.bmt.1703862

[7] Takamura E, Shinozaki K, Hata H, Yukari J, Hori S. Efficacy of autologous serum treatment in patients with severe dry eye. Advances in experimental medicine and biology. 2002;506(Pt B):1247–50. 12614061 10.1007/978-1-4615-0717-8_179

[8] Goto E, Shimmura S, Shimazaki J, Tsubota K. Treatment of superior limbic keratoconjunctivitis by application of autologous serum. Cornea. 2001;20(8):807–10. 11685056 10.1097/00003226-200111000-00006

[9] Rocha EM, Pelegrino FS, de Paiva CS, Vigorito AC, de Souza CA. GVHD dry eyes treated with autologous serum tears. Bone marrow transplantation. 2000;25(10):1101–3. doi: 10.1038/sj.bmt.1702334 10828873 10.1038/sj.bmt.1702334

[10] Tsubota K, Goto E, Shimmura S, Shimazaki J. Treatment of persistent corneal epithelial defect by autologous serum application. Ophthalmology. 1999;106(10):1984–9. doi: 10.1016/S0161-6420(99)90412-8 10519596 10.1016/S0161-6420(99)90412-8

[11] Tsubota K, Goto E, Fujita H, Ono M, Inoue H, Saito I, et al Treatment of dry eye by autologous serum application in Sjogren's syndrome. The British journal of ophthalmology. 1999;83(4):390–5. 10434857 10.1136/bjo.83.4.390 PMC1723012

[12] Ziakas NG, Boboridis KG, Terzidou C, Naoumidi TL, Mikropoulos D, Georgiadou EN, et al Long-term follow up of autologous serum treatment for recurrent corneal erosions. Clinical & experimental ophthalmology. 2010;38(7):683–7. 20456438 10.1111/j.1442-9071.2010.02304.x

[13] Shen EP, Hu FR, Lo SC, Chen YM, Sun YC, Lin CT, et al Comparison of corneal epitheliotrophic capacity among different human blood-derived preparations. Cornea. 2011;30(2):208–14. doi: 10.1097/ICO.0b013e3181eadb67 21045671 10.1097/ICO.0b013e3181eadb67

[14] Soni NG, Jeng BH. Blood-derived topical therapy for ocular surface diseases. British Journal of Ophthalmology. 2015:bjophthalmol-2015-306842. 10.1136/bjophthalmol-2015-306842 26178904

[15] Bradley JC, Simoni J, Bradley RH, McCartney DL, Brown SM. Time- and temperature-dependent stability of growth factor peptides in human autologous serum eye drops. Cornea. 2009;28(2):200–5. doi: 10.1097/ICO.0b013e318186321e 19158565 10.1097/ICO.0b013e318186321e

[16] Kojima T, Higuchi A, Goto E, Matsumoto Y, Dogru M, Tsubota K. Autologous serum eye drops for the treatment of dry eye diseases. Cornea. 2008;27 Suppl 1:S25–30. 18813071 10.1097/ICO.0b013e31817f3a0e

[17] Stenwall PA, Bergstrom M, Seiron P, Sellberg F, Olsson T, Knutson F, et al Improving the anti-inflammatory effect of serum eye drops using allogeneic serum permissive for regulatory T cell induction. Acta ophthalmologica. 2015;93(7):654–7. doi: 10.1111/aos.12801 26178796 10.1111/aos.12801

[18] Atashi F, Jaconi ME, Pittet-Cuenod B, Modarressi A. Autologous platelet-rich plasma: a biological supplement to enhance adipose-derived mesenchymal stem cell expansion. Tissue engineering Part C, Methods. 2015;21(3):253–62. doi: 10.1089/ten.TEC.2014.0206 25025830 10.1089/ten.tec.2014.0206 PMC4346379

[19] Li H, Han Z, Liu D, Zhao P, Liang S, Xu K. Autologous platelet-rich plasma promotes neurogenic differentiation of human adipose-derived stem cells *in vitro*. The International journal of neuroscience. 2013;123(3):184–90. doi: 10.3109/00207454.2012.742077 23126279 10.3109/00207454.2012.742077

[20] Van Pham P, Bui KH, Ngo DQ, Vu NB, Truong NH, Phan NL, et al Activated platelet-rich plasma improves adipose-derived stem cell transplantation efficiency in injured articular cartilage. Stem cell research & therapy. 2013;4(4):91. 23915433 10.1186/scrt277 PMC3854675

[21] Schallmoser K, Bartmann C, Rohde E, Reinisch A, Kashofer K, Stadelmeyer E, et al Human platelet lysate can replace fetal bovine serum for clinical-scale expansion of functional mesenchymal stromal cells. Transfusion. 2007;47(8):1436–46. doi: 10.1111/j.1537-2995.2007.01220.x 17655588 10.1111/j.1537-2995.2007.01220.x

[22] Perez-Ilzarbe M, Diez-Campelo M, Aranda P, Tabera S, Lopez T, del Canizo C, et al Comparison of ex vivo expansion culture conditions of mesenchymal stem cells for human cell therapy. Transfusion. 2009;49(9):1901–10. doi: 10.1111/j.1537-2995.2009.02226.x 19497059 10.1111/j.1537-2995.2009.02226.x

[23] Ben Azouna N, Jenhani F, Regaya Z, Berraeis L, Ben Othman T, Ducrocq E, et al Phenotypical and functional characteristics of mesenchymal stem cells from bone marrow: comparison of culture using different media supplemented with human platelet lysate or fetal bovine serum. Stem cell research & therapy. 2012;3(1):6. 22333342 10.1186/scrt97 PMC3340550

[24] Xia W, Li H, Wang Z, Xu R, Fu Y, Zhang X, et al Human platelet lysate supports ex vivo expansion and enhances osteogenic differentiation of human bone marrow-derived mesenchymal stem cells. Cell Biol Int. 2011;35(6):639–43. doi: 10.1042/CBI20100361 21235529 10.1042/CBI20100361

[25] Mojica-Henshaw MP, Jacobson P, Morris J, Kelley L, Pierce J, Boyer M, et al Serum-converted platelet lysate can substitute for fetal bovine serum in human mesenchymal stromal cell cultures. Cytotherapy. 2013;15(12):1458–68. doi: 10.1016/j.jcyt.2013.06.014 24199591 10.1016/j.jcyt.2013.06.014

[26] Iudicone P, Fioravanti D, Bonanno G, Miceli M, Lavorino C, Totta P, et al Pathogen-free, plasma-poor platelet lysate and expansion of human mesenchymal stem cells. Journal of translational medicine. 2014;12:28 doi: 10.1186/1479-5876-12-28 24467837 10.1186/1479-5876-12-28 PMC3918216

[27] Golebiewska EM, Poole AW. Platelet secretion: From haemostasis to wound healing and beyond. Blood reviews. 2015;29(3):153–62. doi: 10.1016/j.blre.2014.10.003 25468720 10.1016/j.blre.2014.10.003 PMC4452143

[28] Semple JW, Italiano JE Jr., Freedman J. Platelets and the immune continuum. Nature reviews Immunology. 2011;11(4):264–74. doi: 10.1038/nri2956 21436837 10.1038/nri2956

[29] Nurden AT, Nurden P, Sanchez M, Andia I, Anitua E. Platelets and wound healing. Frontiers in bioscience: a journal and virtual library. 2008;13:3532–48. 18508453 10.2741/2947

[30] Huang CJ, Sun YC, Christopher K, Pai AS, Lu CJ, Hu FR, et al Comparison of corneal epitheliotrophic capacities among human platelet lysates and other blood derivatives. PloS One. 2017 2 2;12(2):e0171008 doi: 10.1371/journal.pone.0171008 28152010 10.1371/journal.pone.0171008 PMC5289502

[31] Suri K, Gong HK, Yuan C, Kaufman SC. Human Platelet Lysate as a Replacement for Fetal Bovine Serum in Limbal Stem Cell Therapy. Curr Eye Res. 2016 10;41(10):1266–1273. doi: 10.3109/02713683.2015.1116586 26863375 10.3109/02713683.2015.1116586

[32] Sandri G, Bonferoni MC, Rossi S, Ferrari F, Mori M, Del Fante C, et al Thermosensitive eyedrops containing platelet lysate for the treatment of corneal ulcers. International journal of pharmaceutics. 2012;426(1):1–6. 22248667 10.1016/j.ijpharm.2011.12.059

[33] Geremicca W, Fonte C, Vecchio S. Blood components for topical use in tissue regeneration: evaluation of corneal lesions treated with platelet lysate and considerations on repair mechanisms. Blood Transfus. 2010;8(2):107–12. doi: 10.2450/2009.0091-09 20383304 10.2450/2009.0091-09 PMC2851214

[34] Semeraro F, Forbice E, Braga O, Bova A, Di Salvatore A, Azzolini C. Evaluation of the efficacy of 50% autologous serum eye drops in different ocular surface pathologies. BioMed research international. 2014;2014:826970 doi: 10.1155/2014/826970 25136628 10.1155/2014/826970 PMC4130192

[35] Hussain M, Shtein RM, Sugar A, Soong HK, Woodward MA, DeLoss K, et al Long-term use of autologous serum 50% eye drops for the treatment of dry eye disease. Cornea. 2014;33(12):1245–51. doi: 10.1097/ICO.0000000000000271 25299423 10.1097/ICO.0000000000000271

[36] Lekhanont K, Jongkhajornpong P, Choubtum L, Chuckpaiwong V. Topical 100% serum eye drops for treating corneal epithelial defect after ocular surgery. BioMed research international. 2013;2013:521315 doi: 10.1155/2013/521315 23984378 10.1155/2013/521315 PMC3745890

[37] Aggarwal S, Kheirkhah A, Cavalcanti BM, Cruzat A, Colon C, Brown E, et al Autologous serum tears for treatment of photoallodynia in patients with corneal neuropathy: efficacy and evaluation with *in vivo* confocal microscopy. The ocular surface. 2015;13(3):250–62. doi: 10.1016/j.jtos.2015.01.005 26045233 10.1016/j.jtos.2015.01.005 PMC4499014

[38] Anitua E, Muruzabal F, Tayebba A, Riestra A, Perez VL, Merayo‐Lloves J, et al Autologous serum and plasma rich in growth factors in ophthalmology: preclinical and clinical studies. Acta ophthalmologica. 2015;93(8):e605–e14. doi: 10.1111/aos.12710 25832910 10.1111/aos.12710

[39] Chen Y-M, Hu F-R, Huang J-Y, Shen EP, Tsai T-Y, Chen W-L. The effect of topical autologous serum on graft re-epithelialization after penetrating keratoplasty. American journal of ophthalmology. 2010;150(3):352–9. e2. doi: 10.1016/j.ajo.2010.03.024 20579630 10.1016/j.ajo.2010.03.024

[40] Shen EP, Hu F-R, Lo S-C, Chen Y-M, Sun Y-C, Lin C-T, et al Comparison of corneal epitheliotrophic capacity among different human blood–derived preparations. Cornea. 2011;30(2):208–14. doi: 10.1097/ICO.0b013e3181eadb67 21045671 10.1097/ICO.0b013e3181eadb67

[41] Lu L, Reinach PS, Kao WW-Y. Corneal epithelial wound healing. Experimental Biology and Medicine. 2001;226(7):653–64. 11444101 10.1177/153537020222600711

[42] Montana M, Tabélé C, Curti C, Terme T, Rathelot P, Gensollen S, et al Organic glues or fibrin glues from pooled plasma: efficacy, safety and potential as scaffold delivery systems. J Pharm Pharm Sci. 2012;15(1):124–40. 22365094 10.18433/j39k5h

[43] Nugent RB, Lee GA. Ophthalmic use of blood-derived products. Surv Ophthalmol. 2015; 60(5):406–34. doi: 10.1016/j.survophthal.2015.03.003 26077627 10.1016/j.survophthal.2015.03.003

[44] Herrara M, Solano D, Gómez A, Villalta M, Vargas M, Sánchez A, et al Physicochemical characterization of commercial freeze-dried snake antivenoms. Toxicon. 2017;126:32–37. doi: 10.1016/j.toxicon.2016.12.004 27956243 10.1016/j.toxicon.2016.12.004

[45] Acker JP, Marks DC, Sheffield WP. Quality Assessment of Established and Emerging Blood Components for Transfusion. J Blood Transfus. 2016:4860284 doi: 10.1155/2016/4860284 28070448 10.1155/2016/4860284 PMC5192317

[46] Satirapoj B, Supasyndh O, Choovichian P. A comparative study of efficacy and safety of the lyophilized powder alpha-erythropoietin and the liquid form alpha-erythropoietin for hemoglobin maintenance in patients with hemodialysis treatment. J Med Assoc Thai. 2014;97(9):899–906. 25536706

[47] Imanishi J, Kamiyama K, Iguchi I, Kita M, Sotozono C, Kinoshita S. Growth factors: importance in wound healing and maintenance of transparency of the cornea. Progress in retinal and eye research. 2000;19(1):113–29. 10614683 10.1016/s1350-9462(99)00007-5

[48] Klenkler B, Sheardown H, Jones L. Growth factors in the tear film: role in tissue maintenance, wound healing, and ocular pathology. The ocular surface. 2007;5(3):228–39. 17660896 10.1016/s1542-0124(12)70613-4

[49] Honma Y, Nishida K, Sotozono C, Kinoshita S. Effect of transforming growth factor-beta1 and -beta2 on *in vitro* rabbit corneal epithelial cell proliferation promoted by epidermal growth factor, keratinocyte growth factor, or hepatocyte growth factor. Experimental eye research. 1997;65(3):391–6. doi: 10.1006/exer.1997.0338 9299175 10.1006/exer.1997.0338

[50] Bhowmick NA, Zent R, Ghiassi M, McDonnell M, Moses HL. Integrin beta 1 signaling is necessary for transforming growth factor-beta activation of p38MAPK and epithelial plasticity. J Biol Chem. 2001;276(50):46707–13. doi: 10.1074/jbc.M106176200 11590169 10.1074/jbc.M106176200

[51] Saika S, Kono-Saika S, Ohnishi Y, Sato M, Muragaki Y, Ooshima A, et al Smad3 signaling is required for epithelial-mesenchymal transition of lens epithelium after injury. Am J Pathol. 2004;164(2):651–63. doi: 10.1016/S0002-9440(10)63153-7 14742269 10.1016/S0002-9440(10)63153-7 PMC1602265

[52] Haber M, Cao Z, Panjwani N, Bedenice D, Li WW, Provost PJ. Effects of growth factors (EGF, PDGF-BB and TGF-beta 1) on cultured equine epithelial cells and keratocytes: implications for wound healing. Vet Ophthalmol. 2003;6(3):211–7. 12950652 10.1046/j.1463-5224.2003.00296.x

[53] Shih DT-B, Burnouf T. Preparation, quality criteria, and properties of human blood platelet lysate supplements for ex vivo stem cell expansion. New biotechnology. 2015;32(1):199–211. doi: 10.1016/j.nbt.2014.06.001 24929129 10.1016/j.nbt.2014.06.001 PMC7102808

[54] Guidelines on good manufacturing practices for blood establishments. Annex 4. WHO Tech Rep Series. 2011:148–214

